# Morphometric variation of the *Chiorchis* trematodes, parasitic in the American manatee (*Trichechusmanatus*)

**DOI:** 10.1016/j.ijppaw.2025.101146

**Published:** 2025-10-06

**Authors:** Diana M. Neal, Antonio A. Mignucci-Giannoni

**Affiliations:** a Department of Marine Sciences, University of Puerto Rico, Mayagüez Campus, Puerto Rico, Mayagüez, 00681, USA; b Caribbean Manatee Conservation Center, Inter American University of Puerto Rico, Puerto Rico, Bayamón, 00957, USA; c Conservation Medicine and Ecosystem Health, Ross University School of Veterinary Medicine, Basseterre, Saint Kitts and Nevis

**Keywords:** American manatees, *Trichechus manatus*, Intestinal trematodes, *Chiorchis groschafti*, *Chiorchis fabaceus*

## Abstract

Morphological variation was studied in the adult cladorchiid trematodes *Chiorchis* spp. (Trematoda: Digenea), that parasitize the intestinal tract and caecum of the American manatee (*Trichechus manatus*). Specimens were collected from 22 manatees between 1980 and 1998 in Puerto Rico, Cuba, Dominican Republic, Mexico, and Florida. We found statistically significant differences in analyses of variance in 31 morphological measurements from 284 specimens across different geographical regions, consistent with the existence of two species. Some specimens from Florida corresponded to the description for *Chiorchis fabaceus* Diesing1838, while specimens from the Dominican Republic, Mexico, Puerto Rico, and some from Florida, previously misidentified as *C. fabaceus*, corresponded to the description of *C. groschafti* Coy-Otero1989. The latter supports the distinction of two *Chiorchis* species parasitic on the American manatee. These species differ mainly by the presence or absence of an esophageal bulb, testes shape, position of the genital pore, and shape and distribution of the vitelline follicles.

## Highlights

• Manatees are parasitized by several species of trematodes.
• The most obvious trematodes are intestinal trematodes of the genus *Chiorchis.*
• Two species are known, a freshwater-origin *C*. *fabaceus*, and a marine-origin *C*. *groschafti*, both morphologically different.

## Graphical abstract

**Figure:**
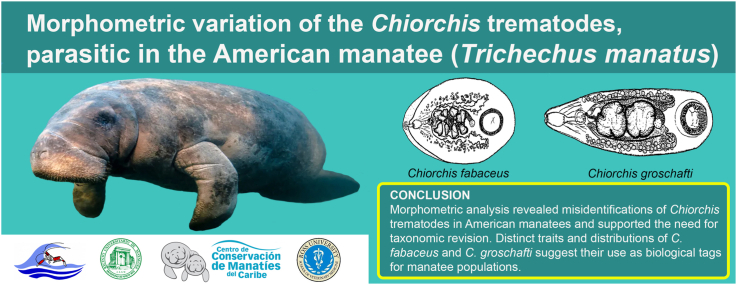

## 1 Introduction

Manatees and dugongs, representatives of the order *Sirenia*, are known to harbor a diverse array of endoparasites (Beck and Forrester, 1988), most of which cause little or no apparent clinical signs of disease (Dierauf, 1990; Geraci and Lounsbury, 1993; Owen et al., 2018). Among sirenians, the American manatee (*Trichechus manatus*), including its two subspecies, the Florida manatee (*T. manatus latirostris*) and the Greater Caribbean manatee (*T.* *manatus manatus*) (Domning and Hayek, 1986; Mignucci-Giannoni et al., 2024), has been extensively studied for its parasitic fauna across its range. Reports of parasitism have emerged from Belize (Sulzner et al., 2012), Brazil (Attademo et al., 2016; Beck and Forrester, 1988; Borges et al., 2011, 2016, 2017; Carvalho et al., 2009), Colombia (Garzón-Gómez, 1997; Vélez et al., 2018, 2019), Cuba (Coy-Otero, 1989), the Dominican Republic (Mignucci-Giannoni et al., 1999b), Florida (Bando et al., 2014; Beck and Forrester, 1988; Blair, 1981; Buergelt et al., 1984; Forrester et al., 1975, 1980; Hutton and Sogandares-Bernal, 1960; Hutton, 1964; Price, 1932; Radhakrishnan and Bradley, 1970; Smith et al., 2016; Sprent, 1980; Wyrosdick et al., 2017), Guyana (Beck and Forrester, 1988), Mexico (Bravo-Hollis and Caballero-Deloya, 1973; Hernández-Olascoaga et al., 2017), and Puerto Rico (Bossart et al., 2012; Colón-Llavina et al., 2009; Mignucci-Giannoni et al., 1999a; Rivera-Pérez et al., 2024a, 2024b, in press; Wyrosdick et al., 2018).

The parasites identified in the American manatee include the ascarid nematode *Heterocheilus tunicatus* (Diesing, 1839), which is located in the stomach and duodenal ampulla; the amoebozoan *Entamoeba* sp. (Casagrandi and Barbagallo, 1895); the protozoans *Eimeria manatus* and *Eimeria nodulosa* (Upton et al., 1989); *Giardia* sp. (Borges et al., 2017); *Toxoplasma gondii* (Nicolle and Manceaux, 1909); and the trematodes *Nudacotyle undicola* and *Moniligerum blairi* (Dailey et al., 1988) found in the intestinal tract, and *Pulmonicola cochleotrema* (Travassos and Vogelsang, 1931) which is found infecting the respiratory tract, mainly in the nasal cavity (Rivera-Pérez et al., 2024a, 2024b). Additionally, two intestinal trematodes—*Chiorchis fabaceus* (Diesing, 1838) and *Chiorchis groschafti* Coy-Otero, 1989—have also been documented parasitizing the caecum and colon. Some researchers have suggested that the latter two helminths associated with sirenians may serve as biological tags to distinguish population structure, track migratory and feeding behaviors, and assess overall health status (Colón-Llavina et al., 2009; Dailey, 1979; Rivera-Pérez et al., 2025 in press).

Despite their relatively common occurrence, questions remain regarding the taxonomic distinction between the two *Chiorchis* species. Earlier studies detailing *Chiorchis* sp. In manatees often failed to provide diagnostic morphological comparisons, leading to assigning all to *C. fabaceus* and ignoring the discovery of *C. groschafti* by Coy-Otero (1989), and thus leading to uncertainty in species differentiation. WoRMS Editorial Board (2025) considered *C. groschafti* a junior subjective synonym of *C. fabaceus*. Here, we review and describe the morphological characteristics of *Chiorchis* specimens across different developmental stages in adult specimens collected from manatees in Cuba, the Dominican Republic, Florida, Mexico, and Puerto Rico, detailing the finding of two distinct species consistent with previously described *C. fabaceus* and *C. groschafti*. This study aims to clarify the taxonomic status of these trematodes based on morphometric evidence and corroborating if any misidentification may come from looking at different developmental stages.

## 2 Materials and methods

### 2.1 Ethics statement

All manatees were found dead, and the entire carcass or its parasites were made available to the authors for this study.

### 2.2 Sample collection

Adult parasites consistent with *Chiorchis* trematodes were collected from the intestinal tracts of 22 deceased American manatees between 1980 and 1998. The manatee carcasses were from Puerto Rico (*n* = 10), the Dominican Republic (*n* = 1), Mexico (*n* = 1), Cuba (*n* = 1), and Florida (*n* = 9), as part of routine salvage and recovery programs in each country (Table 1). The manatees from Florida represented the Florida subspecies (*T. manatus latirostris*), and manatees from Puerto Rico, the Dominican Republic, Mexico, and Cuba belonged to the Greater Caribbean subspecies (*T.* *manatus manatus*). Specimens from Puerto Rico were collected directly by one of the authors, whereas specimens from other countries were provided by the Marine Mammal Pathobiology Laboratory (Florida), the Instituto de Ecología y Sistemática (Cuba), the Acuario Nacional (Dominican Republic), and Proyecto Manatí–ECOSUR (Mexico), all under the appropriate local and international (CITES) permits. During necropsies, the small intestine, caecum, and large intestine were thoroughly examined to collect the helminths, following established protocols (Bonde et al., 1983; Mignucci-Giannoni, 1996).

**Table 1:** American manatees (*Trichechus manatus*) examined for intestinal trematodes, showing field number, date, sex, total length, locality, and cause of death.

*Field number*	*Date*	*Sex*	*Length (cm)*	*Locality*	*Cause of death*
**Cuba**					
CZACC-12-01	Sep 1980	M		Ensenada de la Broa, Ciénaga de Zapata, Matanzas	Capture
**Dominican Republic**					
AN-MAN0	Jun 10, 1995	M	238	Portillo, Las Terrenas, Samaná	Illness
**Florida**					
MNE-9130	Oct 12, 1991	M	328	St. John's River, Jacksonville, Duval	Watercraft collision
MNE-9202	Jan 13, 1992	M	225	Julington Creek, Jacksonville, St. Johns	Undetermined
MSW-9918	Feb 16, 1998	M	243	Matlacha Pass, Matlacha, Lee	Natural
MNE-9818	Apr 27, 1998	M	303	Green Cove Springs, St. John's River, Clay	Undetermined
MEC-9823	May 1, 1998	M	308	Halifax River, Oak Hill, Volusia	Undetermined
MEC-9824	May 2, 1998	M	321	Banana River, Merritt Island, Brevard	Undetermined
MNW-9820	Jul 4, 1998	F	210	Homosassa River, Homosassa, Citrus	Watercraft collision
MSW-9845	Sep 16, 1998	F	276	Myaak River, North Port, Sarasota	Natural
LPZ100942	Jun 3, 1998	M	261	Caloosahatchee River, Ft. Myers, Lee	Ingestion of fishing gear
**Mexico**					
MO-834	Aug 30, 1993	F	270	Expofer, Chetumal, Quintana Roo	Undetermined
**Puerto Rico**					
NEPST150	Apr 11, 1991	F	210	Punta Salinas, Toa Baja	Watercraft collision
NEPST164	May 31, 1991	F	300	Joyuda, Cabo Rojo	Watercraft collision
NEPST186	Apr 18, 1992	M	273	Punta Algodones, Ceiba	Incidental capture
NEPST202	Mar 24, 1993	M	296	Playa Hedionda, Fajardo	Shot
NEPST230	Aug 28, 1993	M	273	Playa Guayanilla, Guayanilla	Illness
NEPST234	Sep 13, 1993	F	311	Playa Corcega, Rincón	Undetermined
NEPST503	Apr 10, 1996	F	207	Sector Pastillo, Juana Díaz	Watercraft collision
NEPST510	Jun 9, 1996	M	229	Punta del Boquerón, Aguada	Watercraft collision
NEPST530	Feb 10, 1997	M	–	Cayo Santiago, Humacao	Illness
NEPST508	Mar 18, 1998	M	277	Ensenada Boca Vieja, Toa Baja	Illness

### 2.3 Preparation of trematodes for examination

**Fig. 1:**
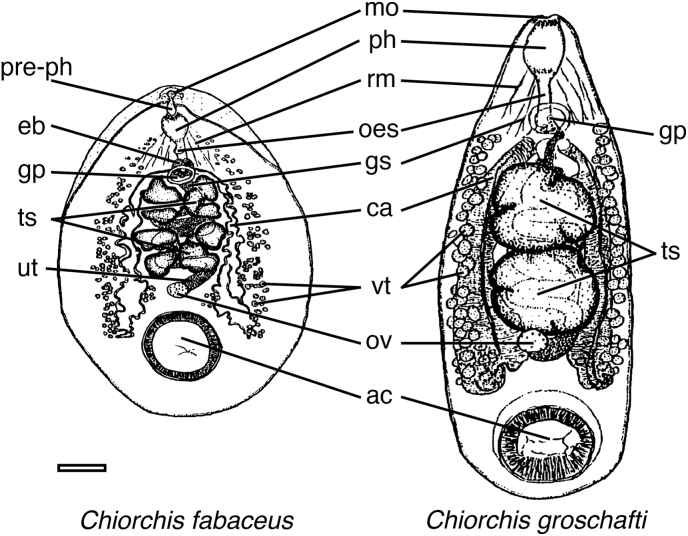
Ventral view of adult *C. fabaceus* and *C. groschafti*, illustrating external and internal anatomical structures. Abbreviations: ac, acetabulum; ca, caecum; oes, oesophagus; eb, esophageal bulb; gp, genital pore; gs, genital sucker; mo, mouth or oral opening; ov, ovary; ph, pharynx; pre-ph, vestiblular pre-pharynx; rm, rectractor muscles of pharynx; ts, testes; ut, uterus; and vt, vitellaria follicles. Scale bar = 1 mm.

Permanent slides were prepared from a subsample of the trematodes collected from each country following the procedures described by Gurr (1962) and Cable (1977). The preparation involved several steps:

• **Fixation and storage**: Trematode specimens were fixed in 10 % neutral buffered formalin. Some were re-fixed in alcohol–formalin–acetic acid (AFA). All samples were then stored in 70 % ethanol. Each vial was labeled with the host's scientific name, collection locality, site of infection, type of fixative used, and necropsy date.
• **Staining and dehydration**: Specimens were initially overstained with Semichon's acetocarmine, rinsed in 70 % ethanol, and destained using 2 % hydrochloric acid in 70 % ethanol. The acid was subsequently removed through serial washes with 70 % ethanol, followed by 80 % grade. Dehydration was performed using a graded series of ethanol (80 %, 95 %, and 100 %).
• **Clearing and mounting**: Fully dehydrated trematodes were cleared using either toluene or xylene after gradual exposure through mixtures with absolute ethanol in the following ratios: 2:1, 1:1, 1:2, and then pure clearing reagent. Specimens were mounted using either Permount or Kleermount media.
• **Measurements and drawings**: A total of 31 morphometric variables were measured for 284 whole-mounted specimens using an eyepiece micrometer (Table 2). Nine measurements were external, and 22 were internal (Fig. 1). Not all structures were visible in every specimen; therefore, sample sizes are reported for each trait. The measurements were reported in millimeters and represent anterior-to-posterior distances. All the specimens were examined from a ventral view except for the excretory pore, which was assessed dorsally. Drawings were created using a drawing tube attached to a Nikon dissecting microscope equipped with incident lighting. Surface features were noted when specimens could not be adequately cleared. Measurement guidelines included: (1) **Genital pore**: measurements include the dimensions of the muscular ring; the anterior distance was measured from the anterior end to the center of the pore; (2) **Acetabulum**: dimensions were obtained from both the internal and external margins of its prominent border; and (3) **Testes, ovaries, and eggs**: maximum length and width values were recorded for these structures.

**Table 2:** Morphological measurements used in the characterization of *Chiorchis* specimens from American manatees (*Trichechus manatus*). Variables include body dimensions and anatomical distances relevant to species differentiation.

	Measurement
1	Body length
2	Body width
3	Anterior to intestinal bifurcation
4	Length paired intestine
5	Anterior to genital pore
6	Length genital pore
7	Width genital pore
8	Anterior to first testis
9	Length of testicular region
10	Length of testicle anterior
11	Width of testicle anterior
12	Length of testicle posterior
13	Width of testicle posterior
14	Anterior to ovary
15	Ovary length
16	Ovary width
17	Anterior to start of vitellaria
18	Length of vitelline region
19	Anterior to acetabulum internal border
20	Anterior to acetabulum external border
21	Acetabulum length (internal border)
22	Acetabulum length (external border)
23	Acetabulum width (internal border)
24	Acetabulum width (external border)
25	Posterior to acetabulum internal border
26	Posterior to acetabulum external border
27	Acetabulum border thickness
28	Length of eggs
29	Width of eggs
30	Anterior to excretory pore
31	Posterior to excretory pore

### 2.4 Data analysis

All morphometric data were standardized as ratios (measurement/body length), except for body length, which was analyzed independently. The distribution of the data was tested using the Kolmogorov–Smirnov goodness-of-fit test (Massey, 1951). Since most variables were not normally distributed and could not be normalized, all statistical analyses used non-parametric methods. Developmental variation, categorized into immature, intermediate, and mature stages, was assessed using the Kruskal-Wallis one-way analysis of variance on ranks (Kruskal and Wallis, 1952). Differences between *C. fabaceus* and *C. groschafti* were tested using the Mann-Whitney *U* test (Sokal and Rohlf, 1995).

## 3 Results

A total of 284 cladorchiid trematodes were recovered and analyzed from intestinal tract of 22 manatees (Table 3). Some of the trematodes were dead upon collection, but most in fresh manatee carcasses were still alive. Specimens from Puerto Rico, as well as voucher samples from the Dominican Republic, Mexico, and some from Florida, had previously been identified as *C*. *fabaceus* (Trematoda: Cladorchiidae) (Mignucci-Giannoni et al., 1999a, 1999b).

**Table 3:** Number of *Chiorchis fabaceus* and *Chiorchis groschafti* specimens measured from 22 manatees, detailing host field number, location within host, number of specimens measured, and trematode species identified.

Host field number	Location in host	Number of specimens measured	Species of *Chiorchis*
**Cuba**			
CZACC-12-01	Small intestine	2	*C. groschafti*
**Dominican Republic**			
AN-MAN0	Colon	5	*C. groschafti*
**Florida**			
MNE-9130	Intestine	6	*C. fabaceus*
MNE-9202	Intestine	18	*C. fabaceus*
MNE-9818	Colon	6	*C. fabaceus*
MEC-9823	Colon	35	*C. fabaceus*
MEC-9824	Proximal colon	6	*C. fabaceus*
MNW-9820	Caecum, proximal colon	10	*C. fabaceus*
MSW-9845	Intestines	3	*C. fabaceus*
MSW-9918	Intestines	5	*C. groschafti*
LPZ100942	Caecum	7	*C. groschafti*
**Mexico**			
MO-834	Intestine	3	*C. groschafti*
**Puerto Rico**			
NEPST150	Caecum	4	*C. groschafti*
NEPST164	Ileum	9	*C. groschafti*
NEPST186	Caecum, colon	5	*C. groschafti*
NEPST202	Caecum, small intestine, colon	9	*C. groschafti*
NEPST230	Small intestine, diverticulum	29	*C. groschafti*
NEPST234	Small intestine	5	*C. groschafti*
NEPST503	Ileum	6	*C. groschafti*
NEPST508	Intestine	50	*C. groschafti*
NEPST510	Intestine	10	*C. groschafti*
NEPST530	Intestine	51	*C. groschafti*

**Total**		**284**	

Upon examination of cleared specimens, 198 trematodes—including all collected from Puerto Rico, the Dominican Republic, Mexico, and select individuals from Florida exhibited consistent morphological features that were distinct from *C. fabaceus*. These characteristics matched those of *C. groschafti*, a species described from a Cuban manatee by Coy-Otero (1989). The remaining 84 specimens, all from Florida manatees, corresponded to the original descriptions of *C. fabaceus* (Diesing, 1838; Fischoeder, 1901). Notably, the two morphotypes were never found within the same individual manatee (Table 3).

A comparative analysis of 31 morphometric characters (measured in millimeters) revealed significant differences between the two morphotypes (Table 4). These results support the distinction between the two species and emphasize the need for a taxonomic revision of the genus *Chiorchis*, focusing on the anatomical differentiation between *C. fabaceus* and *C. groschafti*.

**Table 4:** Summary statistics (mean, standard deviation, and range) for 31 morphological measurements (in mm) of *Chiorchis fabaceus* and *Chiorchis groschafti*. Sample sizes (*n*) vary by measurement. SD = standard deviation.

Measurement	*Chiorchis fabaceus*				*Chiorchis groschafti*			
	*n*	Mean	SD	Range	*n*	Mean	SD	Range
1 Body length	84	5.57	±2.39	2.52–12.72	200	6.27	±2.61	1.28–11.95
2 Body width	84	3.19	±0.94	1.74–6.34	200	3.04	±0.99	0.74–5.71
3 Anterior to intestinal bifurcation	78	1.64	±0.67	0.63–3.77	189	1.59	±0.72	0.39–4.13
4 Length paired intestine	77	2.81	±1.51	1.06–8.30	188	3.27	±1.45	0.32–6.41
5 Anterior to genital pore	63	1.72	±0.66	0.38–3.74	188	1.42	±0.63	0.21–3.56
6 Length genital pore	60	0.38	±0.20	0.06–1.44	177	0.57	±0.19	0.06–0.99
7 Width genital pore	60	0.39	±0.24	0.07–1.73	177	0.65	±0.24	0.08–2.36
8 Anterior to first testis	77	2.01	±0.77	0.96–4.97	182	2.07	±0.89	0.48–0.48
9 Length of testicular region	77	1.39	±0.99	0.38–4.34	178	1.89	±1.03	0.11–4.48
10 Length of testicle anterior	77	0.67	±0.53	0.09–2.16	174	0.97	±0.54	0.03–2.25
11 Width of testicle anterior	77	0.78	±0.56	0.09–2.52	175	1.04	±0.52	0.03–2.12
12 Length of testicle posterior	76	0.66	±0.54	0.09–2.08	176	1.02	±0.60	0.03–2.16
13 Width of testicle posterior	76	0.79	±0.57	0.13–2.56	177	1.12	±0.57	0.04–2.28
14 Anterior to ovary	67	3.63	±1.76	1.46–8.45	169	3.92	±1.81	0.61–8.06
15 Ovary length	64	0.28	±0.17	0.04–0.71	162	0.35	±0.19	0.03–1.89
16 Ovary width	64	0.27	±0.17	0.04–0.79	162	0.38	±0.39	0.03–1.93
17 Anterior to start of vitellaria	50	1.35	±0.58	0.25–2.50	151	1.52	±0.67	0.45–3.42
18 Length of vitelline region	48	3.93	±1.77	2.04–8.49	145	3.81	±1.56	0.85–7.25
19 Ant. To acetabulum internal border	76	3.76	±1.58	1.19–9.35	178	4.81	±2.28	0.18–9.59
20 Ant.to acetabulum external border	49	3.74	±1.83	1.08–9.17	81	4.00	±1.78	0.85–7.71
21 Acetabulum length (internal border)	77	0.88	±0.29	0.34–1.77	156	0.89	±0.49	0.14–2.36
22 Acetabulum length (external border)	46	1.06	±0.41	0.38–2.38	77	0.96	±0.47	0.19–2.49
23 Acetabulum width (internal border)	77	0.96	±0.27	0.56–1.97	163	1.01	±0.51	0.10–2.36
24 Acetabulum width (external border)	47	1.18	±0.34	0.61–2.04	78	1.05	±0.45	0.29–2.38
25 Post. To acetabulum internal border	78	0.69	±0.30	0.18–l.60	158	0.63	±0.35	0.14–1.55
26 Post. To acetabulum external border	44	0.48	±0.23	0.18–1.21	71	0.67	±0.41	0.07–1.57
27 Acetabulum border thickness	46	0.37	±0.21	0.14–1.03	77	0.53	±0.63	0.06–1.51
28 Length eggs	11	0.18	±0.14	0.10–0.52	102	0.10	±0.01	0.07–0.11
29 Width eggs	11	0.16	±0.16	0.06–0.54	101	0.07	±0.01	0.04–0.08
30 Anterior to excretory pore	71	2.15	±0.83	0.85–7.93	84	2.49	±0.95	0.40–5.30
31 Posterior to excretory pore	71	3.07	±l.48	1.09–5.26	84	3.07	±1.23	0.55–4.97

### 3.1 *Chiorchis fabaceus*Diesing (1838); Fischoeder (1901)

Stedman (1889) and Stunkard (1929) provided comprehensive descriptions of the morphology of *C*. *fabaceus* and presented better illustrations than those by Diesing (1838) and Fischoeder (1901). Stedman (1889), with a complete and detailed study of anatomy, focusing on the tegument, parenchyma, and the alimentary tract. However, the excretory and genital systems were only briefly addressed. Despite working with small, mostly immature specimens, Stunkard (1929) contributed additional insights into the descriptions of the excretory and genital systems.

This study presents a detailed description, illustrations, and analysis of the different developmental stages of C. *fabaceus* based on 84 specimens. The specimens were categorized into three classes of sexual development (immature, intermediate, and mature) based on the stage of development of the reproductive organs, and body size.

#### 3.1.1 Immature C. fabaceus

**Fig. 2:**
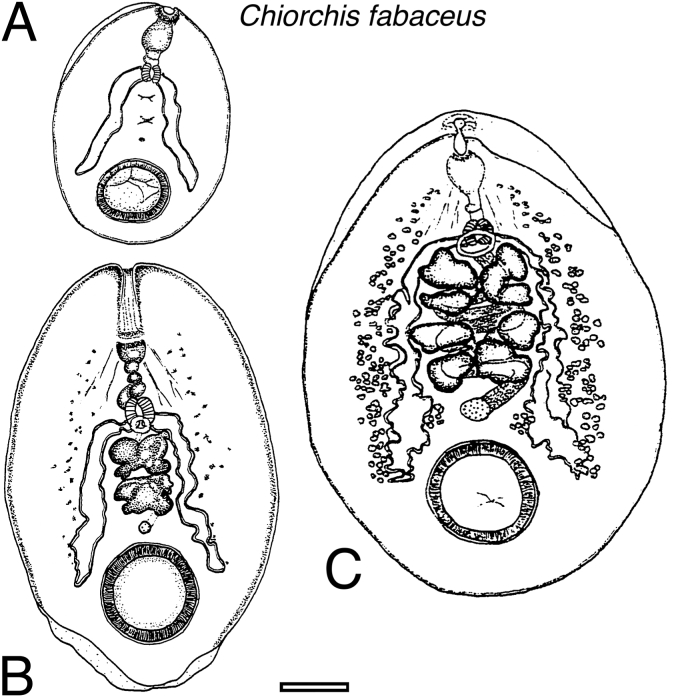
Ventral view of three developmental stages of *Chiorchis fabaceus*: (A) immature, (B) intermediate, and (C) mature. Scale bar = 1 mm.

Forty-one immature specimens from two Florida manatees examined. Body elliptical, flattened ventrally, and convex dorsally (Fig. 2a); 2.51–4.98 mm ($\bar{x}$ = 4.01 mm) by 1.74–3.64 mm ($\bar{x}$ = 2.60 mm). Lateral margins somewhat thinned out to form projecting border. Mouth or oral opening located terminally and ventrally, followed by pre-pharynx that connects with pharynx. Muscle bands radiate posteriorly from the lateral wall of the pharynx. Oesophagus thick-walled, extended backward, becomes ventral, then turns dorsally and again comes dorsally, joining oval esophageal bulb. Alimentary tract bifurcates into thick-walled caeca. Caeca extend straight to level of one-third of acetabulum. Anterior end to cecal bifurcation 0.63–1.78 mm ($\bar{x}$ = 1.26 mm, *n* = 38). Acetabulum ventral with narrow, well-developed border; internal border 0.34–1.41 mm ($\bar{x}$ = 0.77 mm, *n* = 35), and border thickness 0.14–0.72 mm ($\bar{x}$ = 0.31 mm, *n* = 26). Only genital organs distinguishable are testes and ovary, which are not fully developed and not well defined. Testes tandem (Fig. 2a). Anterior testis 0.09–0.78 mm ($\bar{x}$ = 0.32 mm, *n* = 36) and posterior testis 0.09–0.81 mm ($\bar{x}$ = 0.28 mm, *n* = 35). Width of anterior testes 0.09–1.09 mm ($\bar{x}$ = 0.41 mm, *n* = 36) and of posterior testis 0.13–0.94 mm ($\bar{x}$ = 0.40 mm, *n* = 35). Testes exhibit some development of four-branched shape. Ovary a small structure located post-testicular, measuring 0.04–0.34 mm ($\bar{x}$ = 0.15 mm, *n* = 26) by 0.04–0.34 mm ($\bar{x}$ = 0.13 mm, *n* = 26). Seventeen of 41 specimens lack developed genital pore, while 24 had only a small orifice 0.06–0.40 mm ($\bar{x}$ = 0.28 mm) by 0.07–0.44 in width ($\bar{x}$ = 0.28 mm). Only 15 of 41 specimens had vitelline follicles, which were small and without defined margins (Fig. 2a). Excretory pore dorsal, 0.85–3.18 mm ($\bar{x}$ = 2.30 mm, *n* = 39) from anterior end, with strong sphincter. Eggs absent.

#### 3.1.2 Intermediate C. fabaceus

Twenty-four intermediate specimens from three Florida manatees were examined. Body elliptical, flattened on the ventral side, and convex dorsally (Fig. 2b); 5.01–6.63 mm ($\bar{x}$ = 5.38 mm) by 2.96–3.80 mm ($\bar{x}$ = 3.38 mm). Lateral margins thinned to projecting border, posteriorly folded forward. Mouth terminal, ventral; pre-pharynx connecting with pharynx. Muscles bands radiating posteriorly, inserted mid-body wall. Oesophagus thick-walled, extended backward, ventral, then dorsal, joining oval esophageal bulb. Alimentary tract bifurcating into caeca. Caeca slightly sinuous, reaching one-third to one-half acetabulum level; 1.99–3.75 mm ($\bar{x}$ = 2.60 mm, *n* = 22). Cecal walls thickened (Fig. 2b). Acetabulum ventral, well defined; internal border 0.66–1.28 mm ($\bar{x}$ = 0.88 mm, *n* = 23) by 0.78–1.73 mm ($\bar{x}$ = 0.96 mm, *n* = 23). Border thickness 0.20–0.47 mm ($\bar{x}$ = 0.30 mm, *n* = 10). Genital sucker slightly developed; pore ventral to intestinal bifurcation. Testes tandem, deeply four-branched, occupying most intercaecal space. Ovary spherical-oval, ventral, post testicular, medial between posterior testis and acetabulum (Fig. 2b). Vitellaria in 16 of 24 specimens; follicles few, irregular, extending pharynx-acetabulum; 2.38–5.30 mm ($\bar{x}$ = 3.18 mm, *n* = 16). Excretory pore mid-dorsal, 2.81–4.07 mm ($\bar{x}$ = 3.17, *n* = 22), level of posterior testis caudal margin, with strong sphincter. Eggs absent.

#### 3.1.3 Mature C. fabaceus

Nineteen mature specimens from five Florida manatees were examined. Body elliptical, ventrally flattened, dorsally convex (Fig. 2c); 5.06–12.72 mm ($\bar{x}$ = 9.16 mm) by 2.75–6.34 mm in width ($\bar{x}$ = 4.23 mm). Anterior end attenuated in some specimens due to contracted pharyngeal muscle bands. Muscle bands anchored mid-body wall. Lateral margins forming strong projecting border. Mouth terminal, ventral; pre-pharynx connecting pharynx. Pharynx oval with two posterior diverticula. Oesophagus thick-walled, backward then ventral, dorsal, and ventral again, joining muscular oval esophageal bulb. Distance anterior end–esophageal bulb 1.21–3.77 mm ($\bar{x}$ = 2.46 mm, *n* = 18). Alimentary tract bifurcating into caeca. Caeca straight to slightly sinuous, extending one-third–one-half acetabulum level; 2.43–8.30 mm ($\bar{x}$ = 5.17 mm, *n* = 17). Cecal walls thick. Acetabulum ventral, prominent border; border thickness 0.10–0.17 mm ($\bar{x}$ = 0.12 mm, *n* = 10). External border length 1.08–2.38 mm ($\bar{x}$ = 1.60 mm, *n* = 10). Ovary spherical–oval, 0.22–0.71 mm ($\bar{x}$ = 0.47 mm, *n* = 19) by 0.27–0.79 mm (‾x = 0.47 mm, n = 19); post-testicular, medial between posterior testis and acetabulum, $\bar{x}$ = 5.88 mm from anterior end. Oviduct from dorsal anterior/lateral margin, loosely coiled intercaecal, turning ventral between anterior testis and esophageal bulb, merging with ejaculatory duct, forming common genital pore. Genital sucker weak; pore ventral, posterior to intestinal bifurcation, 1.37–3.74 mm ($\bar{x}$ = 2.43 mm, *n* = 17) from anterior end. Testes tandem, deeply four-branched, lobes swollen; anterior testis 0.52–2.16 mm by 0.65–2.52 mm; posterior wider. Testes occupy full intercaecal space (1.24–4.34 mm, $\bar{x}$ = 2.79 mm, *n* = 19); branches overlapping caeca. Uterus with thin-shelled eggs, $\bar{x}$ = 0.12 mm by $\bar{x}$ = 0.09 mm. Vitellaria scattered, extending pharynx–posterior caecum, 2.47–8.49 mm, 2.47–8.49 mm, ($\bar{x}$ = 5.42 mm, *n* = 19); follicles oval–irregular, some intercaecal. Excretory pore mid-dorsal at caudal margin of posterior testis, with strong sphincter.

#### 3.1.4 Variation in C. fabaceus

The classification of developmental stages was clearly defined by the presence of eggs and the development of the sexual structures. The all-immature specimens had a body length less than 5.00 mm. Some intermediate specimens, ranging from 5.01 to 6.63 mm, overlapped in length values with mature specimens but did not contain eggs. However, three trematodes measuring 5.05, 5.50, and 6.31 mm, under the higher length value for intermediate specimens, were already sexually mature. The ratio between the body width and body length of the different developmental stages showed that immature and intermediate specimens were not significantly different (*P* = 0.185). Still, both these stages were significantly different (*P* = 2.04 × 10^−10^) relative to the mature stages. The body widths of the first two stages ($\bar{x}$ = 0.65 and 0.63, respectively) were wider than the mature stage ($\bar{x}$ = 0.48) relative to the total length of the trematodes. The immature and intermediate specimens maintained a proportion of the body width and the body length that corresponded to an oval shape. When they reached maturity, the specimens became more elongated with higher long-axis values than widths. The three developmental stages possessed a wide space, filled with parenchyma on each lateral side. The internal organs are embedded in this tissue and confined at the center of the body (Fig. 2). All the specimens had a distinguishable long vestibular pre-pharynx before the pharynx and a sinuate oesophagus through their developmental stages. There were no significant differences (*P* = 0.79) between the level that the caecum reach ($\bar{x}$ = 2.93 mm, *n* = 26) and the acetabulum position ($\bar{x}$ = 3.11 mm, *n* = 27) among immature specimens. The caecum of immature trematodes extended to the anterior margin of the acetabulum. In contrast, intermediate and mature trematodes showed a greater extension of the caecum, reaching one third to the center of the acetabulum.

The mature trematodes presented a significant difference (*P* = <0.01 and *P* = 0.04, respectively) between the level that the caecum reach and the acetabulum position. Clear morphological variability was observed in the sinuosity of the caecum and the stages of development. The sinuosity of the caecum increases in the intermediate and mature stages. Nevertheless, some mature specimens have caeca with the sinuosity restricted only to the ends. The thickness of the acetabulum border with respect to body length was not significantly different (*P* = 0.16) throughout the growth stages of this species. However, the length of the acetabulum in proportion to the body length showed significant differences (*P* = <0.001) in the three stages. The size of the acetabulum in proportion to the body size of immature trematodes is larger ($\bar{x}$ = 0.19, *n* = 35) than intermediate ($\bar{x}$ = 0.15 mm, *n* = 24) and mature ($\bar{x}$ = 0.12 mm, *n* = 19) specimens. The testes are H-shaped for immature and intermediate specimens and deeply tetra-lobed for mature trematodes. The space between the testes decreases when the trematodes reach sexual maturity. The position of the testes is intercaecal, but in some specimens, the anterior lobules overlapped the caecum. The ovaries tend to be more oval than rounded, located post-testicular and centered between the caecum. The position of the genital pore and the caecum bifurcation level in the three stages of development were not significantly different (*P* = 0.83). However, mature specimens tended to have the genital pore positioned more posteriorly relative to the cecal bifurcation. Immature stages rarely exhibited vitellaria follicles. If they are present, they are small and limited to the center of the cecal zone. As the size of trematodes increases, the follicles extend forward to the pharynx level and posteriorly to the ends of the caecum and into the intercaecal areas.

### 3.2 *Chiorchis groschafti*Coy-Otero (1989)

The description provided by Coy-Otero (1989) based on 25 specimens lacks information, and the illustrations are poorly elaborated. The pharynxwas described as subterminal, and the esophageal bulb was noted to be located at the level of the bifurcation of the caecum. However, the published illustrations showed that the position of the pharynxwas terminal, and there was no clearly defined esophageal bulb. In addition, the structures such as the uterus, vesicle, excretory pore, and other structures were not described. Two hundred specimens were grouped into distinct sexual development classes (immature, intermediate, and mature) based on the development stage of the reproductive organs, the presence of eggs and the body size. *C. groschafti* has small sexually mature specimens with eggs, as well as some medium-size specimens with well-developed sexual organs, but without eggs.

#### 3.2.1 Immature C. groschafti

**Fig. 3:**
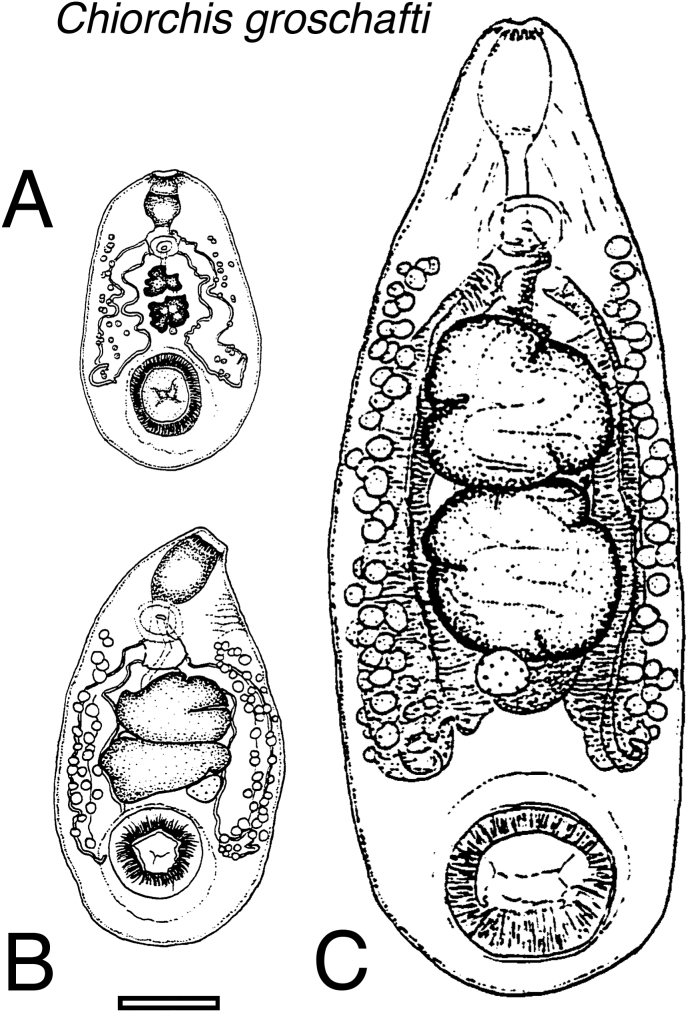
Ventral view of three developmental stages of *Chiorchis groschafti*: (A) immature, (B) intermediate, and (C) mature. Scale bar = 1 mm.

Seven immature specimens from one manatee from Puerto Rico were examined. Body conical–pyriform, ventrally flattened, dorsally convex, no lateral border (Fig. 3a); 1.28–2.06 mm ($\bar{x}$ = 1.52 mm) by 0.74–1.12 mm ($\bar{x}$ = 0.89 mm). Mouth terminal; short vestibular pre-pharynx to pharynx. Pharynx with posteriorly radiating muscle bands. Oesophagus short, straight, thick-walled, posteriorly expanded, no esophageal bulb. Alimentary tract bifurcates into thick-walled caeca. Caeca straight–sinuate, reaching acetabulum anterior level, ends turning ventral then anterior. Distance anterior end–caecum posterior margin 0.79–1.33 mm ($\bar{x}$ = 1.03 mm, *n* = 7). Testes small, tandem, separated; ovary post-testicular, centered intercaecal. Genital pore present, unopened externally. Acetabulum ventral, well developed; internal border 0.14–0.24 mm ($\bar{x}$ = 0.21 mm, *n* = 7); border thickness 0.06–0.24 mm (‾x = 0.14 mm, n = 6). Vitellaria and eggs absent. Excretory pore dorsal, sphincter absent.

#### 3.2.2 Intermediate C. groschafti

Five intermediate specimens from one manatee from Puerto Rico were examined. Body conical–pyriform, ventrally flattened, dorsally convex, no lateral border (Fig. 3b); 1.69–2.15 mm ($\bar{x}$ = 1.91 mm) by 0.90–1.50 mm ($\bar{x}$ = 1.13 mm). Mouth terminal; vestibular pre-pharynx to pharynx. Pharynx with posteriorly radiating muscle bands. Oesophagus short, straight, thick-walled, posteriorly expanded, no esophageal bulb. Alimentary tract bifurcates into thick-walled caeca. Caeca straight–sinuate, surrounding testes and ovary, projecting laterally to acetabulum, ends ventral then anterior. Distance anterior end–caecum posterior margin 0.75–1.77 mm ($\bar{x}$ = 1.31 mm, *n* = 5). Sexual organs well defined but small, anteriorly positioned. Testes tandem, contiguous, oval–irregular with indentations, not branched. Ovary oval–rounded, immediately post-testicular, centered intercaecal. Genital pore present in all specimens, 0.06–0.23 mm ($\bar{x}$ = 0.12 mm) by 0.07–0.18 mm ($\bar{x}$ = 0.13 mm). Acetabulum ventral, well developed; internal border 0.17–0.44 mm ($\bar{x}$ = 0.27 mm, *n* = 5); border thickness 0.17–0.23 mm ($\bar{x}$ = 0.18 mm, *n* = 4). Vitellaria rare, only in one specimen; follicles small, oval–round, scattered in cecal area, not intercaecal. Eggs absent. Excretory pore dorsal, sphincter absent.

#### 3.2.3 Mature C. groschafti

One hundred eighty-eight mature specimens examined: one from Dominican Republic, one from Cuba, 10 from Puerto Rico, three from Florida. Body subcylindrical, anterior attenuated, ventrally concave, dorsally convex, no lateral border (Fig. 3c); 2.42–11.95 mm ($\bar{x}$ = 6.56 mm) by 1.57–5.71 mm ($\bar{x}$ = 3.16 mm). Mouth terminal; vestibular pre-pharynx to pharynx. Pharynx with posteriorly radiating muscle bands. Oesophagus long, thick-walled, slightly sinuate, ventral mid-length, posteriorly expanded, no esophageal bulb. Alimentary tract bifurcates into thick-walled caeca. Caeca slightly sinuate, terminating near acetabulum anterior level; distance anterior end–caecum posterior margin 1.68–10.54 mm ($\bar{x}$ = 5.11 mm, *n* = 176). Sexual organs occupying one-third body length. Testes tandem, contiguous, oval–irregular, indented, not branched; 0.61–4.48 mm ($\bar{x}$ = 2.00 mm, *n* = 167), filling intercaecal space. Ovary oval–rounded, post-testicular, centered intercaecal; displaced slightly lateral in 37 specimens. Uterus filled with thin-shelled eggs, $\bar{x}$ = 0.09 mm by $\bar{x}$ = 0.07 mm. Uterus arising from dorsal anterior/lateral ovary, coiling intercaecal, dorsal to testes, turning ventral between anterior testis and cecal bifurcation. Vasa efferentia uniting anterior to testis; seminal vesicle thin-walled, coiled; followed by pars musculosa enclosed in prostatic cells; ejaculatory duct joining metraterm, forming hermaphroditic structure. Genital sucker weak, with parenchymal muscle ring. Genital pore anterior to cecal bifurcation, 0.21–3.56 mm ($\bar{x}$ = 1.46 mm, *n* = 179). Vitellaria scattered follicles, round–oval, extending pharynx–posterior caecum or beyond, 0.99–7.25 mm ($\bar{x}$ = 3.83 mm, *n* = 144); dorsal to caeca, not intercaecal. Excretory pore mid-dorsal at caudal margin of posterior testis, sphincter absent.

#### 3.2.4 Variation in C. groschafti

Adults of *C. groschafti* reach sexual maturity soon after their last juvenile stage. Immature and intermediate stages were found only in specimens with a body length of less than 2.15 mm. The adult stage differs from immature specimens primarily by the increased definition of the testes and ovary, the presence of an open genital pore, and a greater number of vitellaria follicles. Although mature specimens were producing eggs, other organs showed morphological variability as the trematode reached larger sizes. Therefore, the morphological variability of *C. groschafti* was based on specimens clustered in size classes of small type (1.28–4.56 mm in length) (including immature, intermediate, and small mature specimens), medium type (4.66–7.99 mm in length) and large type (8.11–11.95 mm in length). All specimens of *C. groschafti* examined exhibited a short vestibular pre-pharynx before the pharynx; the oesophagus was shorter in small specimens in relation to pharynx size. In medium and large specimens, there was an increase in the length of the oesophagus, which is slightly sinuate (observed in the sagittal section). In the three types, the posterior end of the oesophagus expands giving origin to each caecum. In small and medium specimens, the caecum extend straight or slightly sinuate, surrounding the testes and the ovary. Adjacent to the ovary, the caecum projects laterally, reaching toward the acetabulum. The ends of the caecum turn ventrally and then anteriorly. This position changed for larger specimens, which have caeca straight with sinuous ends but not reaching the acetabulum. The position of the caecum in relation to the acetabulum differed significantly (*P* = 5.79 × 10^−5^) among size classes. Small and medium trematodes showed the caecum reaching the anterior level of the acetabulum (*P* = 0.46), whereas in large specimens, the caecum ended anteriorly to the acetabulum region. In all size classes, the position of the genital pore was always anterior to cecal bifurcation, with no significant differences observed among their ratios (*P* = 0.12). The position of the testes in small specimens was anterior compared to those in the other two size classes. The shape of the testes varied greatly. Small and medium types had highly indented testes without a distinguishable lobed or branched pattern. In contrast, some specimens in both size-types exhibited testes more oval with a tendency to be four-lobed. Medium-sized specimens showed an indentation in each testis lobule. In contrast, large specimens had more rounded testes with slight sagittal and transversal constrictions, forming four lobules without deep depressions. The ovary was oval to round. It was immediately post-testicular and centered between the caecum. The vitellaria follicles in small specimens were confined to the length of the caecum, but in medium and large specimens, the follicles extended anterior to the genital pore. The follicles were either oval or round and always extended over the caecum and dorsally but never intercaecal. The uterus was more visible when it was filled with eggs, passing dorsally to the testes in firmly coiled ducts and turned ventrally between the anterior testis and cecal bifurcation, reaching the genital pore. Some mature specimens contained thin-shelled eggs in the genital sucker. Fibrous tissues to which the muscular fibers are attached surround the ventral sucker. The thickness of the acetabulum border, in relation to body length, was not significantly different (*P* = 0.81) throughout the growth of these trematode. However, the acetabulum length in proportion to the body length was significantly different (*P* = <0.001) when comparing small specimens with medium and large ones. No significant differences (*P* = 0.58) were noted between the last two. The acetabulum ratio in small trematodes was higher ($\bar{x}$ = 0.23, *n* = 25) than in medium ($\bar{x}$ = 0.17 mm, *n* = 43) and large ($\bar{x}$ = 0.15 mm, *n* = 9) specimens. The length of the acetabulum exhibited allometric growth in relation to the body length. The excretory pore moves anteriorly through their body development.

## 4 Discussion

### 4.1 *Chiorchis* species descriptions

*Chiorchis fabaceus* was first described by Diesing (1838, 1839) from Amazonian manatees (*Trichechus inunguis*), although the account was brief and largely descriptive. Later studies expanded on its morphology: Stedman (1889) detailed the tegument, parenchyma, and alimentary tract, while Stunkard (1929) contributed observations on the excretory and genital systems. Fischoeder (1901) established the genus *Chiorchis*, and subsequent authors reported specimens from *T. manatus* and the African manatee ( *T. senegalensis*), often attributing minor differences to immaturity (Stunkard, 1929; Travassos, 1934; Travassos et al., 1969). Many early synonymizations (e.g., *Schizamphistoma manati*, Sokoloff and Caballero, 1932) were later incorporated into *C. fabaceus* (Yamaguti, 1971).

In the late 20th century, new records extended the species’ distribution in Florida and the Caribbean (Beck and Forrester, 1988; Mignucci-Giannoni et al., 1999a, 1999b), though most identifications defaulted to *C. fabaceus*. Coy-Otero (1989), however, described *C. groschafti* from a Cuban manatee, based on its smaller size, absence of an esophageal bulb, and unlobed testes. This distinction was not widely recognized, and specimens from Puerto Rico, the Dominican Republic, and Mexico continued to be labeled as *C. fabaceus*.

Our review of historical material and the present morphometric analysis support the validity of *C. groschafti* as a distinct species, and highlight the need for continued taxonomic work. While previous descriptions emphasized subtle or inconsistent traits, our study refines the distinguishing features between the two taxa and provides a framework for future research on manatee parasites.

### 4.2 Morphological variation between *C. fabaceus* and *C. groschafti*

Traditionally, features such as the presence of a genital sucker, esophageal bulb, and testis or acetabulum morphology have been used to differentiate cladorchiid trematodes (Yamaguti, 1971). Our results confirm that these criteria are reliable, but also demonstrate that *C. fabaceus* and *C. groschafti* differ in a broader suite of characters.

Superficial similarities have long led to confusion, with *C. groschafti* specimens from Puerto Rico, the Dominican Republic, and Florida often misidentified as *C. fabaceus* (Beck and Forrester, 1988; Mignucci-Giannoni et al., 1999a, 1999b). More recent work has corrected this (Colón-Llavina et al., 2009). Our morphometric analysis confirms and expands these observations, highlighting consistent and significant differences across specimens.

The most diagnostic features can be summarized as follows:

*Body shape and size*: *C. fabaceus* is larger, elliptical, with a pronounced lateral border; *C. groschafti* is smaller, subcylindrical, and lacks a lateral border.

*Mouth and vestibular pre-pharynx*: In *C. fabaceus*, the mouth is terminal and ventrally directed with a prominent vestibular pre-pharynx; in *C. groschafti*, the vestibular pre-pharynx is reduced.

*Pharynx musculature*: Present in both species, likely a generic rather than specific trait.

*Oesophagus*: *C. fabaceus* has a distinct sinuate (“S”-shaped), elongated oesophagus; *C. groschafti* has a shorter, straighter oesophagus.

*Esophageal bulb*: Present and well developed in *C. fabaceus*; completely absent in *C. groschafti*.

*Cecal extension relative to acetabulum*: In *C. fabaceus*, the caeca may extend anterior to the acetabular level; in *C. groschafti*, the caeca remain posterior and never approach the acetabulum.

*Testes*: Deeply tetra-lobed in *C. fabaceus*; rounded to irregular, sometimes slightly indented, in *C. groschafti*.

*Genital pore*: More anterior in *C. groschafti*; more ventral or posterior in *C. fabaceus*.

*Vitellaria*: *C. fabaceus* shows scattered, sometimes intercaecal follicles; *C. groschafti* exhibits exclusively extra-cecal follicles.

*Sexual maturity*: *C. groschafti* reaches maturity at smaller body sizes, possibly reflecting different reproductive strategies.

Taken together, these differences support recognition of *C. groschafti* as a valid species. While some individual characters may overlap, their combined pattern provides a clear distinction between the two taxa. Importantly, these traits were consistent across multiple geographic regions, reinforcing the robustness of the diagnosis.

### 4.3 Revised key to species of *Chiorchis*

The recognition of *C*. *groschafti* as a new species described in 1989, along with the observed interspecific morphological variability in *C. fabaceus*, necessitates revisions to the identification and classification key for the family Paramphistomidae. The following revised key is more detailed than that of Yamaguti (1971), emphasizing the distinguishing morphological characters identified in the present study.

Body elliptical, flattened dorso-ventral, lateral border with pronounced edge. Acetabulum small (1/7 of the total body length). Esophageal bulb well defined, genital sucker ventral or slightly posterior to the cecal bifurcation. Vitellaria follicles far from lateral borders, and some follicles intercecal. Testes deeply tetra-lobed. Caecum reach the level of 1/3 to 1/2 of the acetabulum length ………………*C*. *fabaceus*

Body sub-cylindrical, lateral edge without a pronounced border. Acetabulum prominent (1/6 of the total body length). Esophageal bulb absent. Genital sucker anterior to cecal bifurcation, vitellaria follicles close to lateral borders, extra-cecal. Testes rounded to irregular in shape or slightly four-lobed. Caecum usually do not extend to the anterior border of the acetabulum, but never more than 1/4 of the acetabulum length …………………………………………………*C*. *groschafti*

## 5 Conclusions

Our study demonstrates that *Chiorchis fabaceus* and *C. groschafti* are morphologically distinct parasites of the American manatee. Careful examination of museum and reference material revealed that many earlier identifications were erroneous, highlighting the need for rigorous taxonomic work. Both species were never found within the same individual: *C. fabaceus* was recovered exclusively from Florida manatees, while *C. groschafti* occurred in the insular Caribbean and Mexico, with Florida being the only region where both species were present. This geographic segregation suggests differences in intermediate host distribution: the absence of *C. fabaceus* in the insular Caribbean indicates that its intermediate host(s) may be restricted to continental freshwater systems, while the widespread occurrence of *C. groschafti* implies that its host(s) are associated with marine or coastal environments in both continental and insular habitats (Rivera-Pérez et al., 2025 in press).

These findings clarify the status of two long-confused trematodes and emphasize their potential as biological tags for manatee population studies, contributing to our understanding of distribution, movements, and feeding ecology (Rivera-Pérez et al., 2025 in press). To strengthen species identification and classification within this group, additional *Chiorchis* samples from other American manatee localities (e.g., Brazil, Venezuela, Guyanas, Colombia, Panama, Mexico) and from other manatee species (e.g., African and Amazonian manatees) should be examined. Given the evolutionary divergence of the African manatee from the American lineage 1.5–3.3 million years ago (J. Vélez-Juarbe, pers. comm.; de Souza et al., 2021), and its wide marine, riverine, and lacustrine distribution, it would not be surprising if African manatees harbor distinct or even novel *Chiorchis* species.

Future work should also investigate the co-occurrence of *Chiorchis* spp. With other helminths infecting manatees. In addition, molecular analyses are needed to further support the systematic placement of these intestinal trematodes within the Paramphistomidae. Combined with morphometric evidence, such studies will expand our understanding of parasite diversity, evolution, and their role in manatee health and ecology.

## CRediT authorship contribution statement

**Diana M. Neal:** Writing – review & editing, Writing – original draft, Visualization, Validation, Methodology, Formal analysis, Data curation. **Antonio A. Mignucci-Giannoni:** Writing – review & editing, Writing – original draft, Visualization, Validation, Supervision, Resources, Project administration, Data curation, Conceptualization.

## Declaration of competing interest

We confirm that there are no conflicts of interest to declare.
